# A Treatise on Diseases of the Joints

**Published:** 1861-10

**Authors:** 


### Book Notices.

A Treatise on Diseases of the Joints. By Richard Barwell, F. R. C. S., Assistant Surgeon Charing-Cross Hospital, etc. Illustrated by Engravings on Wood. Philadelphia: Blanchard & Lea. 1861.

Barwell's name will be recognized by the reader familiar with the periodical literature of the profession, as one of the most earnest and original monographists of the past half-dozen years. His contributions to the knowledge of the physiological and pathological anatomy of the tissues of joints,—elaborate and valuable in themselves,—were, however, but preliminary steps in the accomplishment of his object, and "were not undertaken," as he says, in his modest preface, "so much from love for that sort of work as from a perception that certain links must be supplied, certain entanglements unravelled, and error, if any existed, corrected."

The present volume forms a land-mark, so to speak, in the improvements which have been made in this branch of surgery; clearly and practically arranging and defining the acquirements and limits of our present knowledge, the records of which, have existed, heretofore, only in scattered essays and isolated papers, mixed up with somewhat of downright error, not a little that was empirical, and much that needed the further test of time and enlarged experience to warrant its acceptance. The work is divided into two parts, of nine chapters each; the first chapter, devoted to the physiological anatomy of the joints, is valuable and interesting, aside from its clear, succinct treatment, on account of the peculiar views of the author on some points, differing from preceding writers. Among these, his description of the articular lamella (pp. 25, 26,) views which met much opposition when first broached, in 1859;—his exposition of the arrangement of the synovial membrane upon cartilage (p.p. 29,30;) his puncture of Weber's synovial vacuum theory; and his reconciliation of Quekett's and Bowman's conflicting representations of the yellow fibrous tissue, are some of the more important. It is hardly necessary to observe that his statements are as well sustained and as carefully, though boldly made, as in the original papers, the substance of which this chapter embodies.

Chapter II. is an exhaustive treatment of *Acute Synovitis*, sixteen pages on its pathology, seven on the symptoms, and ten on the treatment,—abundantly supplied with clinical illustrations. Mr. BARWELL "cordially commends the value of" free incisions into suppurating joints, where the wound is small, or there is no wound,—not simply "to allow shreds of cartilage to escape" as advanced by Gay, but because "a joint once suppurated, has lost that sensitiveness to the contact of air which it normally possesses; it is an abscess, and one cause of the great constitutional disturbance produced by the disease, is confinement of matter deep among bones and tough fibrous structure. Therefore, if a *depending* part of the joint can be in any way reached, it should be wisely incised."

Partly from its bearing upon the chronic form, *Acute Rheumatism*, though not legitimately within the surgical province, is next treated of; partly from the above reason, but also, and not less, we suspect, "because a very high authority [Dr. Todd], has recorded his opinion, that the local affection of the joints is not inflammatory." *En passant*, we may mention a trait which impresses us not less forcibly than our author's sturdy Saxon sense, plain, direct, and straightforward,—that is his national combative proclivity. Not that we mean by this to imply that he seeks for argument and contradiction; but whenever he meets what he conceives to be error, he straightway attacks it, and usually, as in the present instance, with pronounced success.

*Pyarthrosis*,—purulent absorption, pyæmia, or a half-dozen other synonyms—is the subject of the fourth chapter, embracing a consideration of the various theories of its obscure pathology, and a résumé of the unsatisfactory results of experiments. BARWELL considers the disease largely resultant from a peculiar zymotic influence, affecting the blood, and relies on quinine mainly in its treatment. The chapter on *Strumous Synovitis*, which is the next in order, is one of, if not *the* most interesting in the volume. No sketch can do anything like justice either to the chapter or the book itself,—a fitting review of which we do not attempt, but hasten to give the headings of the remaining chapters: Chapter VI.—*Rheumatic Synovitis.* Chapter VII.—*On Some Other Forms of Chronic Synovitis*—syphilitic, gouty, simple. Chapter VIII.—*Hydrarthrosis*,—pathology, symptoms, treatment, cases. Chapter IX.—*On Loose Cartilages in the Joints.* Part second is devoted to the consideration of *Diseases Commencing in the Bone*,—the first part of the volume having embraced, as will be seen by the above titles, only those diseases commencing in the synovial membranes and sub-synovial tissue. For greater accuracy and clearness, Mr. BARWELL designates inflammation of joint-bones *Articular Osteitis*, and divides it into two kinds,—the acute and the chronic, after the German method. Chapter X. treats of the first division, and the succeeding chapter, of *Strumous Articular Osteitis*,—an essay rivalling in value and completeness anything we have ever read on the subject. *Chronic Rheumatic Osteitis*, *Inflammation and Degeneration of Cartilages*, *Hip-Joint Disease*, *Affections of Synovial Sheaths and Bursæ in the Neighborhood of Joints*, *Restoration of Mobility and Conformity to Crippled Joints*, and the

*Removal of Diseased Joints*, form, respectively, the subjects of the remaining chapters.

Where there is so much that is meritorious, we have no wish to weaken our approval by censure or fault-finding. And yet we could wish that the distinguished author had shown less cockneyism in his citation of authorities, made, as they are, to the almost complete exclusion of the American. Chapter X. betrays an ignorance of "the most elaborate and complete work on Surgery ever published in any country,"* only equalled by the almost total ignoring, in chapter XIV. (Hip Joint disease,) of such American authorities as March, Harris, Davis, Sayre, Andrews, Taylor and others, and their hip-joint splints,—to which, by the way, his own is decidedly inferior.

In succeeding editions of the work, which we have no doubt its intrinsic worth will soon necessitate, it will be the duty of the American publishers to submit its pages to the supervision of competent hands, who shall supply the deficiencies, and correct the errors we allude to. Its present handsome typographical appearance, and accurate illustrations, reflect credit on the enterprise and good taste of the publishers.

**London Lancet*, and *Dublin Quarterly Journal of Medical Science*,—reviews of Prof. S. D. Gross' *System of Surgery*, 1861.

